# Acute hepatitis E virus superinfection increases mortality in patients with cirrhosis

**DOI:** 10.1186/s12879-022-07050-w

**Published:** 2022-01-18

**Authors:** Jung Woo Choi, Ho Jin Son, Sang Soo Lee, Hankyu Jeon, Jin-Kyu Cho, Hee Jin Kim, Ra Ri Cha, Jae Min Lee, Hyun Jin Kim, Woon Tae Jung, Ok-Jae Lee

**Affiliations:** 1 Department of Internal Medicine, Gyeongsang National University School of Medicine, Gyeongsang National University Hospital, Jinju, Republic of Korea; 2 Institute of Health Sciences, Gyeongsang National University, Jinju, Republic of Korea; 3 Department of Internal Medicine, Gyeongsang National University Changwon Hospital, Changwon, Republic of Korea; 4 Department of Surgery, Gyeongsang National University School of Medicine, Gyeongsang National University Hospital, Jinju, Republic of Korea

**Keywords:** Hepatitis E virus, Chronic liver disease, Cirrhosis, Acute-on-chronic liver failure, Mortality

## Abstract

**Background:**

Although acute hepatitis E is not fatal in healthy individuals, it is unclear whether hepatitis E superinfection increases the mortality in patients with pre-existing liver disease. Thus, we investigated the prognosis of patients with acute hepatitis E according to their cirrhosis diagnosis, and the prognosis according to the development of acute-on-chronic liver failure (ACLF) in patients with cirrhosis and chronic liver disease (CLD).

**Methods:**

This study included 74 consecutive patients who were diagnosed with acute viral hepatitis E between January 2007 and December 2019. Of them, 39 patients without CLD, 13 patients with non-cirrhotic CLD, and 22 patients with cirrhotic CLD were analyzed.

**Results:**

Among the 74 patients with HEV infection, 7 (9.5%) died within 180 days: 5 with underlying cirrhosis (71.4%) and 2 without cirrhosis (28.6%). The 180-day mortality was significant higher for patients with cirrhosis than for patients without cirrhosis (22.7% vs. 3.8%, *P* = 0.013). The age- and sex-adjusted proportional-hazard model revealed an approximately eightfold increase in the 180-day mortality risk in patients with cirrhosis compared to patients without cirrhosis. In addition, development of hepatitis E virus-related ACLF due to acute liver function deterioration in patients with pre-existing CLD or cirrhosis worsened the 180-day mortality rate.

**Conclusions:**

Our findings suggest that the acute hepatitis E mortality rate was low in healthy individuals but higher in patients with cirrhosis, and especially high in those with ACLF.

**Supplementary Information:**

The online version contains supplementary material available at 10.1186/s12879-022-07050-w.

## Background

The burden of disease attributed to hepatitis E virus (HEV) genotypes 1 and 2 is estimated to be approximately 20 million incident infections, resulting in 70,000 deaths per year among developing countries [1]. From the seroprevalence data, two million infections of HEV genotypes 3 and 4 have also been estimated in developed countries [2–4].

In the past, HEV infection was perceived as a disease limited to developing countries, since HEV genotypes 1 and 2 spread via the fecal–oral route. However, locally acquired HEV infections attributed to genotypes 3 and 4 have emerged among patients in most high-income countries who have not travelled to developing regions [5]. The incidence of hepatitis E infection varies both between and within countries and also changes over time. For example, the incidence of hepatitis E infection is particularly more common in France than in other European countries; however, it also varies between 0.3% and 4.6% within France itself [6]. In high-income Asian countries such as Korea and Japan, locally acquired hepatitis E is an emerging infection. HEV is still recognized as a rare cause of acute viral hepatitis in Korea (2%) [7], but it is no longer considered rare in the southeast of Korea (20%) [8].

Hepatitis E superinfection is a concern in patients with chronic liver disease (CLD). In particular, acute hepatitis E can be a potential precipitating factor for acute-on-chronic liver failure (ACLF). Small case series from developing countries have reported that superinfection of hepatitis E in patients with pre-existing CLD is associated with high mortality [9–11]. Most previous studies of HEV-related ACLF have been conducted in low- and middle-income countries in Asia and Africa [12], and have reported short-term mortality rates ranging between 0 and 67%, with a median of 34% [11]. In contrast, there are few studies on HEV-related ACLF in Asian and Western high-income countries, where genotypes 3 and 4 predominate. In one European study of 343 patients with decompensated CLD, only 11 patients had acute HEV infection and three of those (27%) died within 6 months [13].

In Korea, several case series of acute hepatitis E genotypes 3 and 4 have been reported [8, 14–16]. However, there are no reports on the prognosis of locally acquired HEV infection in CLD in Korea, a high-income country in Asia. Therefore, the present study aimed to compare the prognosis of patients with acute hepatitis E according to their cirrhosis diagnosis in Korea. In addition, we compared the prognosis according to the occurrence of HEV-related ACLF in patients with cirrhosis and CLD.

## Patients and methods

### Study population

A total of 77 consecutive patients who were clinically diagnosed with acute hepatitis E infection at the Gyeongsang National University Changwon Hospital and Gyeongsang National University Hospital from January 2007 to December 2019 were identified. The exclusion criteria were as follows: (1) Non-Korean foreigners (n = 2), (2) normal value of aminotransferase (n = 1), (3) human immunodeficiency virus co-infection (n = 0), (4) pregnant women (n = 0), and (5) age < 18 years (n = 0). Among the remaining 74 patients with acute hepatitis E, 52 individuals without cirrhosis and 22 with cirrhosis were finally analyzed. The study was approved by the Institutional Review Boards of Gyeongsang National University Changwon Hospital (IRB File No. 2018-07-009) and Gyeongsang National University Hospital (IRB File No. 2014-04-028) and was conducted in accordance with the principles of the Declaration of Helsinki (1975). The need for informed consent was waived owing to the retrospective design of this study, as determined by both the Institutional Review Boards.

### Data gathering and definition

Clinical information on concomitant diseases such as hypertension, diabetes, chronic kidney disease, and malignancy were retrieved from the electronic health records. Laboratory data including the levels of white blood cells, hemoglobin, platelets, total bilirubin, aspartate aminotransferase, alanine aminotransferase, creatinine, albumin, sodium, and international normalized ratio, were collected. Patients’ histories were reviewed to identify the exposure to risk factors including uncooked or undercooked meat consumption, sexual exposure, travel history, alcohol consumption, previous blood transfusions, and clinical symptoms.

Acute hepatitis E infection was defined as positive detection of anti-HEV IgM [Dia.Pro, Milan, Italy] and/or positive HEV RNA in real-time reverse transcription PCR assay, as previously described, and acute illness with typical symptoms of acute hepatitis and/or abnormal liver function tests [8].

Liver cirrhosis was defined as evidence of portal hypertension, manifested as splenomegaly, ascites, varices, or hepatic encephalopathy, and compatible imaging findings accompanied by thrombocytopenia (< 100,000/µL). CLD was defined as the persistent deterioration of liver function over 6 months with the presence of one or more of the following diseases: alcoholic liver disease, chronic hepatitis B or C, autoimmune hepatitis, and cirrhosis. To investigate the effect of ACLF development on the mortality in patients with HEV infection, we used two widely accepted definitions of ACLF by the Asia–Pacific Association for the Study of Liver (APASL) and the European Association for the Study of the Liver (EASL). According to the EASL-chronic liver failure criteria for ACLF (EASL-ACLF), ACLF was defined as the development of acute deterioration (acute onset of overt ascites, hepatic encephalopathy, gastrointestinal hemorrhage, and bacterial infection) in the patients of cirrhosis who were at risk of organ failure and high short-term mortality based on the Consortium Acute-on-Chronic Liver Failure in Cirrhosis study [17]. Organ failure was defined according to the Chronic Liver Failure-Organ Failure score [18], and involved the following: liver failure (total bilirubin level of ≥ 12 mg/dL), kidney failure (serum creatinine level of ≥ 2.0 mg/dL and/or requiring renal support therapy), cerebral failure (grade III or IV hepatic encephalopathy based on West Haven criteria), coagulation failure (international normalized ratio > 2.5), circulation failure (treatment with vasoconstrictors to maintain arterial blood pressure or inotropes to improve cardiac output), and respiratory failure (PaO_2_/FiO_2_ ≤ 200 or SpO_2_/FiO_2_ ≤ 214). APASL-ACLF was defined as an acute hepatic insult manifesting as jaundice (serum bilirubin ≥ 5 mg/dL) and coagulation dysfunction (international normalized ratio ≥ 1.5) complicated within 4 weeks by overt ascites and/or hepatic encephalopathy in patients with CLD [19]. Both the patients diagnosed with ACLF at admission and those who developed ACLF during their hospital stay were included in the analysis. Alcoholic hepatitis was diagnosed based on the clinical criteria [20].

### Statistics

Continuous variables were summarized as medians (interquartile ranges) and categorical variables as frequencies (percentages). Comparisons between the groups were performed using the Mann–Whitney U test for continuous variables, and Fisher’s exact test for categorical variables. Survival rates in all the patients classified by cirrhosis diagnosis and in the patients with cirrhosis and/or CLD classified by ACLF were estimated by the Kaplan–Meier method and compared using the log-rank test. We used a Cox proportional hazards regression model to examine the predictors associated with 180-day mortality. The risk was expressed as a hazard ratio (HR) and 95% confidence interval (CI). The association between cirrhosis and 180-day mortality was evaluated via multivariate analysis after adjusting for age and sex. A *P*-value < 0.05 was considered statistically significant for all analyses. Data handling and statistical operations were performed using PASW Statistics, version 18 (SPSS Inc., Chicago, IL, USA).

## Results

### Patient characteristics

A total of 74 patients were diagnosed with acute hepatitis E based on serological investigations and clinical presentations over the course of the study period. Of these 74 patients, 22 (29.7%) patients with cirrhosis and 52 (70.3%) without cirrhosis were analyzed (Table 1). The proportion of diabetes was higher in patients with cirrhosis (36.4%) than in those without (5.8%, *P* = 0.001). However, there was no significant difference in age, sex, alcohol consumption, hypertension, chronic kidney disease, and malignancy between the two groups.

**Table 1:** Baseline characteristics of patients with acute hepatitis E infection (n = 74)

	Total	No cirrhosis	Cirrhosis	*P*
No	74 (100%)	52 (70.3%)	22 (29.7%)	
Age, year	56.0 (42.8–68.0)	55.5 (40.3–68.8)	56.5 (47.0–68.0)	0.670
Male gender	50 (67.6%)	34 (65.4%)	16 (72.7%)	0.597
Diabetes	11 (14.9%)	3 (5.8%)	8 (36.4%)	0.001
Alcohol > 40 g/day	20 (27.0%)	11 (21.2%)	9 (40.9%)	0.080
Hypertension	13 (17.6%)	9 (17.3%)	4 (18.2%)	1.000
CKD	2 (2.7%)	2 (3.8%)	0 (0%)	1.000
Malignancy	12 (16.2%)	9 (17.3%)	3 (13.6%)	1.000
Clinical symptoms				
Jaundice	33 (44.6%)	20 (38.5%)	13 (59.1%)	0.128
Fatigue	18 (24.3%)	13 (25.0%)	5 (22.7%)	0.543
Nausea/vomiting	10 (13.5%)	10 (19.2%)	0 (0%)	0.028
Fever	14 (18.9%)	13 (25.0%)	1 (4.5%)	0.052
Abdominal pain	16 (21.6%)	12 (23.1%)	4 (18.2%)	0.764
No symptom	14 (18.9%)	10 (19.2%)	4 (18.2%)	1.000
Undercooked meat	14 (18.9%)	10 (19.2%)	4 (18.2%)	1.000
Laboratory data				
WBC, × 10^9^/L	6.1 (5.1–9.6)	6.0 (4.9–9.5)	6.3 (5.1–10.9)	0.736
Hemoglobin, g/dL	13.4 (11.7–14.8)	13.6 (12.0–15.2)	13.0 (11.1–14.4)	0.274
Platelet, × 10^9^/L	177.5 (116.5–266.3)	206.5 (142.0–276.8)	117.0 (83.3–159.8)	< 0.001
AST, U/L	257.0 (72.5–868.3)	329.0 (109.5–852.0)	168.5 (55.0–1288.0)	0.232
ALT, U/L	347.5 (89.3–959.3)	395.5 (129.5–1032.8)	90.0 (24.5–585.0)	0.009
Creatinine, mg/dL	0.82 (0.65–0.91)	0.80 (0.65–0.89)	0.84 (0.65–0.93)	0.452
Bilirubin, mg/dL	4.3 (1.3–8.7)	2.2 (0.9–8.2)	6.8 (2.5–15.7)	0.008
Albumin, g/dL	3.7 (3.4–4.1)	3.9 (3.5–4.2)	3.5 (2.8–3.8)	0.003
Sodium, mmol/L	136.9 (134.4–139.5)	138.0 (135.0–139.8)	134.1 (131.4–137.3)	0.004
PT-INR	1.13 (1.00–1.44)	1.06 (0.99–1.16)	1.51 (1.19–2.02)	< 0.001

*CKD* chronic kidney disease, *WBC* white blood cell, *AST* aspartate transaminase, *ALT* alanine transaminase, *PT-INR* prothrombin time- international normalized ratio

*P*: Mann–Whitney U-test and Chi-squared test

Data are presented as the median (interquartile range) for continuous data and percentages for categorical data

Jaundice (44.6%) was the most frequent symptom in all patients, followed by fatigue (24.3%), abdominal pain (21.6%), fever (18.9%), and nausea/vomiting (13.5%). Among the patients with acute HEV infection, 14 (18.9%) were asymptomatic. Interestingly, a significant number of patients with HEV infection consumed undercooked meat (18.9%).

Patients with cirrhosis had significantly lower platelet, alanine aminotransferase, albumin, and sodium levels compared to patients without cirrhosis. Moreover, patients with cirrhosis had significantly higher bilirubin levels and a higher international normalized ratio compared to patients without cirrhosis (Additional file 1: Fig. S1).

### Mortality and its associated factors

**Fig. 1:**
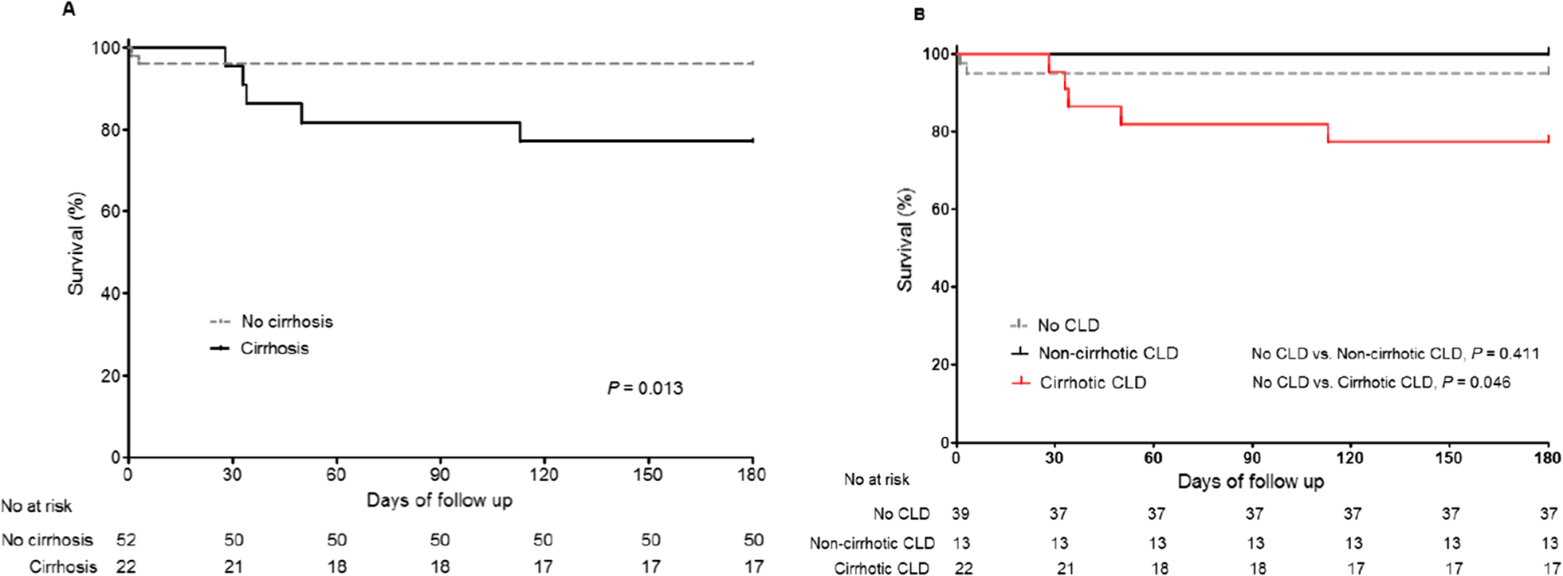
Kaplan–Meier survival curves for 180-days mortality stratified by underlying cirrhosis in patients with hepatitis E infection (n = 74)

Among patients with HEV infection, 7 (9.5%) died within 180 days (Additional file 2: Table S1). One patient underwent liver transplantation 181 days following his diagnosis with HEV infection. The cause of death in all patients was liver failure. Ribavirin was used in the treatment of HEV infection in 2 patients, and steroid was used in 3 patients owing to severe alcoholic hepatitis and cholestasis. To determine whether underlying cirrhosis was associated with mortality, we compared the 180-day mortality between HEV patients with and without cirrhosis. Of the 7 patients who died within 180 days, 5 had underlying cirrhosis (71.4%) and 2 did not (28.6%). The 180-day mortality rate was significantly higher for patients with cirrhosis than for those without (22.7% vs. 3.8%, *P* = 0.013) (Fig. 1A) (Additional file 2: Table S2).

Of the 74 patients with acute hepatitis E, 39 patients without CLD, 13 patients with non-cirrhotic CLD, and 22 patients with cirrhotic CLD were analyzed using the Kaplan–Meier method (Fig. 1B). The 180-day HEV mortality rate was higher for patients with cirrhotic CLD than for patients without CLD (22.7% vs. 5.1%, *P* = 0.046); however it was similar for patients with non-cirrhotic CLD and patients without CLD (0% vs. 5.1%, *P* = 0.411).

The unadjusted proportional-hazards model demonstrated that patients with cirrhosis had an increased risk of 180-day mortality compared to patients without cirrhosis (HR = 6.151, 95% CI = 1.192–31.726). After adjustment for age and sex, underlying cirrhosis significantly increased the risk of 180-day mortality (HR = 8.111, 95% CI = 1.432–45.937) (Additional file 2: Table S3).

### EASL-ACLF in patients with cirrhosis

**Fig. 2:**
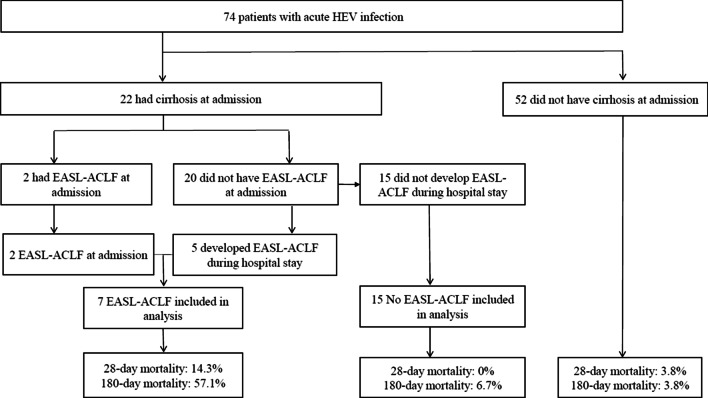
Flow sheet according to EASL-ACLF development

**Fig. 3:**
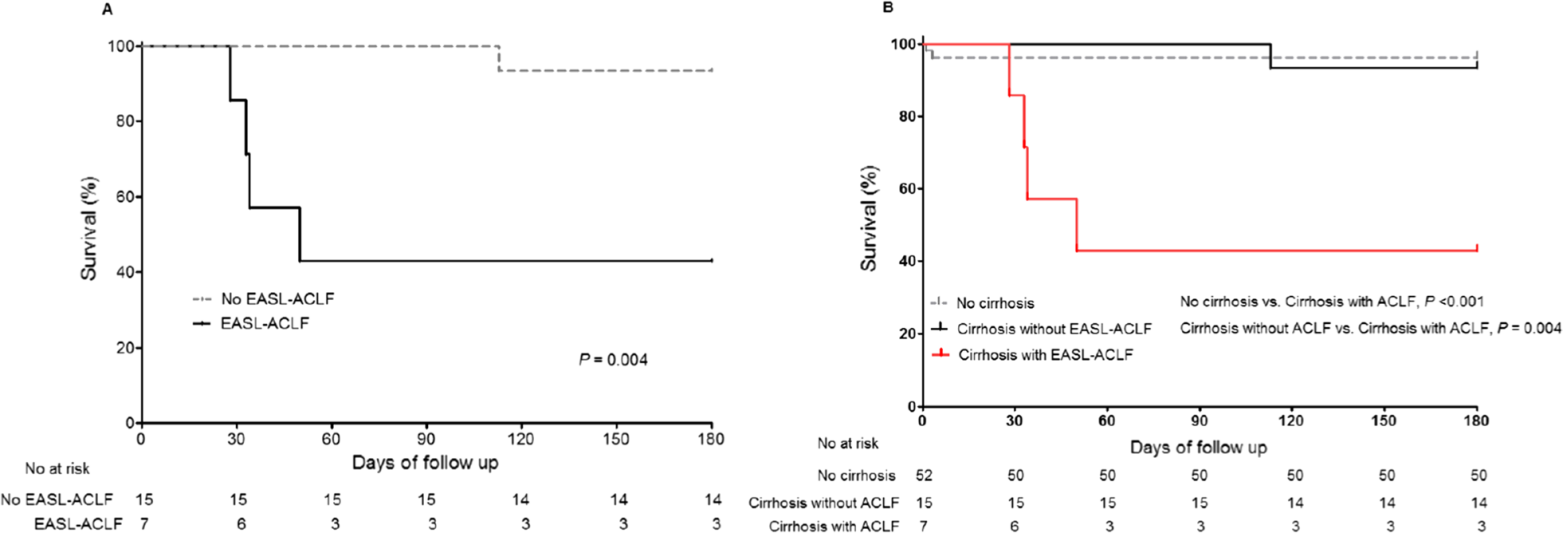
Kaplan–Meier survival curves for 180-days mortality **A** according to EASL-ACLF in patients with cirrhosis (n = 22) and **B** according to cirrhosis and EASL-ACLF in total patients (n = 74)

To investigate the association between EASL-ACLF and HEV infection, Chronic Liver Failure-Organ Failure scores were measured in 22 patients with underlying cirrhosis. Of these patients, 7 (31.8%) were identified as having EASL-ACLF: two were diagnosed at admission (Fig. 2) (Additional file 2: Table S4) and 5 developed EASL-ACLF during their hospital stay. Mortality at 28 days and 180 days was 3.8% and 3.8% for patients without cirrhosis, 0% and 6.7% for those with cirrhosis without EASL-ACLF, and 14.3% and 57.1% for those with cirrhosis with EASL-ACLF, respectively. The 180-day mortality of HEV infection in patients with cirrhosis (n = 22) was significant higher for patients with EASL-ACLF than for patients without EASL-ACLF (*P* = 0.004) (Fig. 3A). The 180-day mortality rate was higher for patients with cirrhosis with EASL-ACLF than for patients without cirrhosis (*P* < 0.001) and for those with cirrhosis without EASL-ACLF (*P* = 0.004) (Fig. 3B).

### APASL-ACLF in patients with CLD

**Fig. 4:**
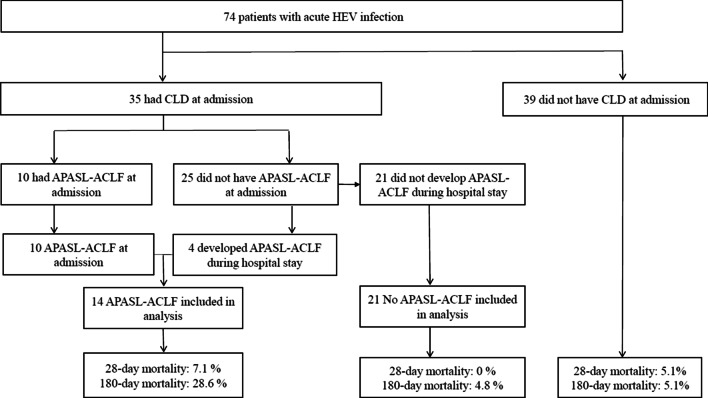
Flow sheet according to APASL-ACLF development

**Fig. 5:**
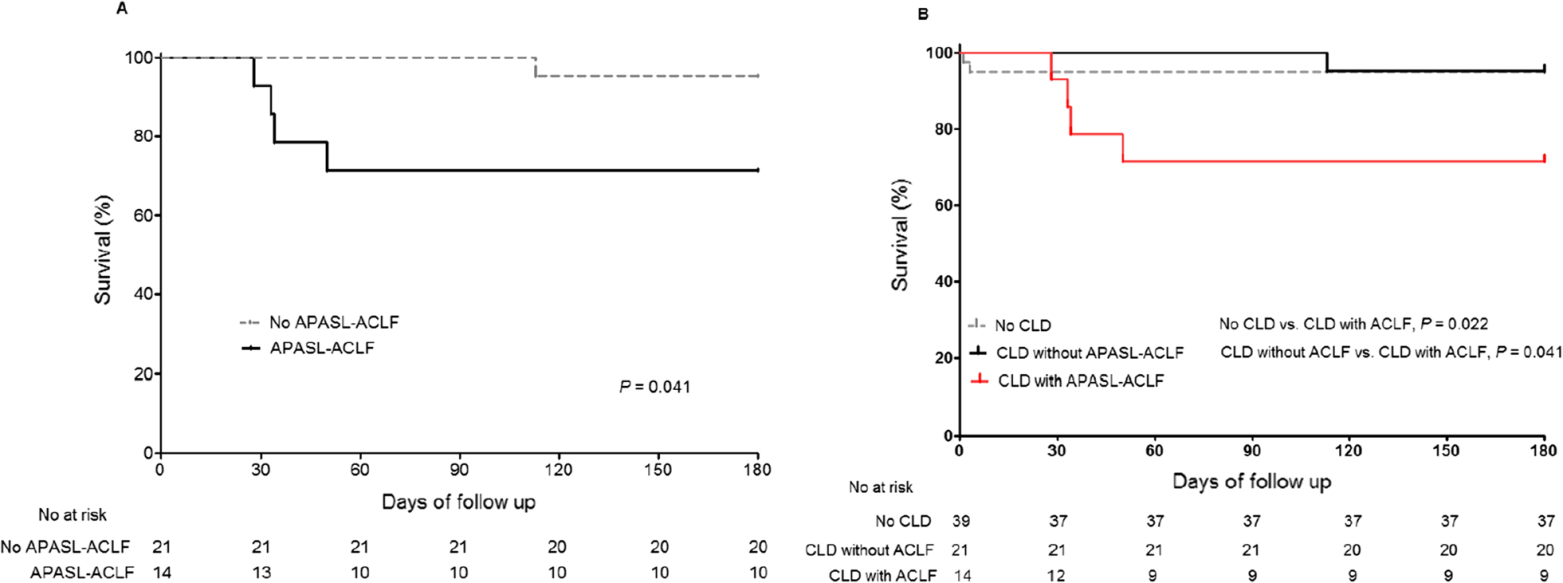
Kaplan–Meier survival curves for 180-days mortality **A** according to APASL-ACLF in patients with cirrhosis (n = 35) and **B** according to cirrhosis and APASL-ACLF in total patients (n = 74)

In a total of 35 HEV-infected patients with underlying CLD, 14 were identified as having APASL-ACLF (40%): ten patients were diagnosed with APASL-ACLF at admission and 4 developed APASL-ACLF during their hospital stay (Fig. 4). Mortality at 28 days and 180 days was 5.1% and 5.1% for patients without CLD, 0% and 4.8% for those with CLD without APASL-ACLF, and 7.1% and 28.6% for those with CLD with APASL-ACLF, respectively. The 180-day mortality of HEV-infected patients with CLD (n = 35) was significant higher for patients with APASL-ACLF than for patients without APASL-ACLF (*P* = 0.041) (Fig. 5A). Furthermore, 180-day mortality was higher for patients with CLD with APASL-ACLF than for patients without CLD (*P* = 0.022) and for those with CLD without APASL-ACLF (*P* = 0.041) (Fig. 5B).

## Discussion

We set out to investigate not only whether the presence of underlying cirrhosis affects the survival rate of patients with acute hepatitis E, but also to elucidate the impact of ACLF development in HEV-infected patients with cirrhosis and CLD. In this observational cohort study of 74 Korean patients with acute HEV infection, mortality at 180 days was 9.5% (3.8% for patients without cirrhosis vs. 22.7% with cirrhosis, *P* = 0.013). The age- and sex-adjusted proportional-hazard model revealed that patients with underlying cirrhosis had a significantly increased risk of mortality compared to patients without cirrhosis (HR = 8.111). The development of ACLF (according to EASL and APASL criteria) had a significant effect on the 180-day mortality rate in patients with HEV infection.

A peculiar result of this study was the approximately the eightfold increase in the risk of the 180-day mortality in patients with cirrhosis compared to patients without cirrhosis. Previous studies conducted in Asia found a high 12-month mortality rate approaching 70% in HEV-infected patients with underlying CLD [9, 21]. To date, the impact of HEV infection on CLD/cirrhosis in Europe has not been studied as well as in Asian countries. Data from small European studies revealed that autochthonous HEV-infected patients (3 patients in the UK and 7 in France) with pre-existing CLD had an approximate mortality rate of 70% [22, 23]. However, recent data from 11 patients with decompensated CLD in UK/France showed three (27%) died within 180 days of presentation [13]. All studies investigating the mortality of HEV-infected patients with underlying CLD/cirrhosis in Europe and Asia had a very small sample size. In two Chinese studies, hepatitis E viral superinfection in patients with chronic hepatitis B resulted in more severe clinical outcomes [24, 25]. The epidemiology of HEV is changing in China, where the previously dominant genotype 1 has become less common while the zoonotic genotypes 3 and 4 are now the most commonly observed in middle-aged Chinese men [26]. A very recent study with a large sample size of Chinese patients, including 56 patients with cirrhotic CLD, 47 with non-cirrhotic CLD, and 124 with no CLD, showed that superinfection with HEV in patients with cirrhotic CLD had a worse outcome than HEV-infected patients with non-cirrhotic CLD or without CLD [27]. However, this study did not provide the mortality rate of patients with HEV infection.

A recent study from Hong Kong found that coexisting chronic hepatitis B was associated with an increased 30-day liver-related mortality (5.3% vs. 1.5%, *P* = 0.018) in patients with acute HEV infection [28]. In contrast, the most recently published study from China demonstrated that underlying end-stage liver disease, not pure hepatitis B infection, increases mortality (9.6% vs. 1.7%, *P* < 0.001) in patients with acute hepatitis E [29]. In our study on 22 patients with underlying cirrhosis, the 180-day mortality rate for hepatitis E was 22.7%. Patients with cirrhosis, but not those with CLD, had a significantly higher mortality rate than those without underlying liver disease.

However, there are several limitations in all studies, including ours, namely involving symptomatic HEV infected patients, a fraction of the vast majority of hepatitis E patients, usually asymptomatic. Therefore, studies of HEV patients, excluding asymptomatic patients, inevitably overestimated the mortality and did not accurately represent the clinical outcomes of the asymptomatic patients. In addition, the frequency of patients with CLD and cirrhosis was overestimated compared to that in the general population. Moreover, recently, a retrospective cohort study on Taiwanese patients with chronic hepatitis B infection following HEV superinfection, including asymptomatic patients, defined HEV superinfection as HEV-IgG seroconversion during the study period [30]; they demonstrated that acute HEV superinfection increased the risk of liver-related mortality in chronic hepatitis B patients, especially in those with cirrhosis (1-year mortality rate of 35.7%).

The mortality of HEV-related ACLF varies widely in different Asian studies. Studies of HEV genotype 1 infection reported short-term mortality rates between 0 and 67% for cases of ACLF (most studies used the APASL-ACLF criteria) [11]. All the case series on patients with HEV-related ACLF patients in Europe also had a very small sample size (maximum sample size of 11 patients) [31]; moreover, these studies did not apply the ACLF criteria of EASL or APASL. Our study, investigating HEV-related ACLF by applying the EASL and APASL criteria, has a larger sample size than previous European studies. Our study revealed a higher 180-day mortality rate in ACLF patients diagnosed by EASL (57.1%) and APASL (28.6%) criteria than those without ACLF and without underlying liver disease. These results are comparable to the high mortality rate of 15–25% reported in HEV-infected pregnant women [32]. Our findings suggest that both worsened pre-existing liver status and the development of organ failure play an important role in the prognosis of patients with acute HEV infection. The PREDICT study, a European, multicenter, prospective, observational study, showed that established bacterial infection and severe alcoholic hepatitis accounted for almost all cases of AD and ACLF [33]. In our study, ACLF was related to bacterial infection in 2 patients (28.6%) and alcoholic hepatitis in 3 patients (42.9%).

Although acute hepatitis E has been considered a rare cause of acute viral hepatitis in Korea, the reported IgG anti-HEV seroprevalence data (17–27%) suggest that the prevalence of HEV infection may be underestimated [34]. In a previous study conducted in the southeastern region of Korea, we reported that acute hepatitis E is no longer a rare cause of acute viral hepatitis. However, there have been no reports on mortality of patients with acute hepatitis E in Korea. We found that the mortality rate of acute hepatitis E was low in the general population without underlying liver disease, but high in patients with cirrhosis, and particularly in those with ACLF.

Our study has certain limitations. First, clinical symptoms of acute hepatitis and the presence of IgM anti-HEV (not HEV-PCR) were used to diagnose acute hepatitis E in most cases. Another limitation of our study is the retrospective nature of the data collection, which could lead to recall bias, such as in the alcohol drinking data collection. In our study, most patients were clinically diagnosed with cirrhosis; however, this was not confirmed histologically. Nevertheless, this is a relatively large-scale study, which systematically reported the association between mortality and the development of ACLF using both EASL and APASL criteria in patients with cirrhosis/CLD.

## Conclusion

Our study revealed that underlying cirrhosis contributes to the high risk of mortality among patients with acute HEV infection. In addition, the development of ACLF owing to acute liver function deterioration in patients with CLD/cirrhosis resulted in higher mortality.

## Supplementary Information

**Additional file 1. Fig. S1.**

**Additional file 2: Table S1. **Comparison of variables between survivors and non-survivors in HEV-infected patients (n = 74). **Table S2.** Comparison of variables between survivors and non-survivors in patients with cirrhosis (n = 22). **Table S3. **Predictive factors of 180-day mortality (n = 74). **Table S4.** Outcome in 7 patients with acute-on-chronic liver failure.

## Data Availability

The datasets generated and/or analyzed during the current study are not publicly available due to ethical and confidentiality reasons but are available from the corresponding author on reasonable request under the Gyeongsang National University Changwon Hospital and Gyeongsang National University Hospital Ethics Committee’s approval. The data that support the findings of this study are available on request to the correspondence author. (Sang Soo Lee, Email:3939lee@naver.com).
