# Supplementary figures and images for: Acute hepatitis E virus superinfection increases mortality in patients with cirrhosis
Source: BMC Infect Dis. 2022 Jan 18;22:62. doi: 10.1186/s12879-022-07050-w (PMC8767750; doi:10.1186/s12879-022-07050-w)

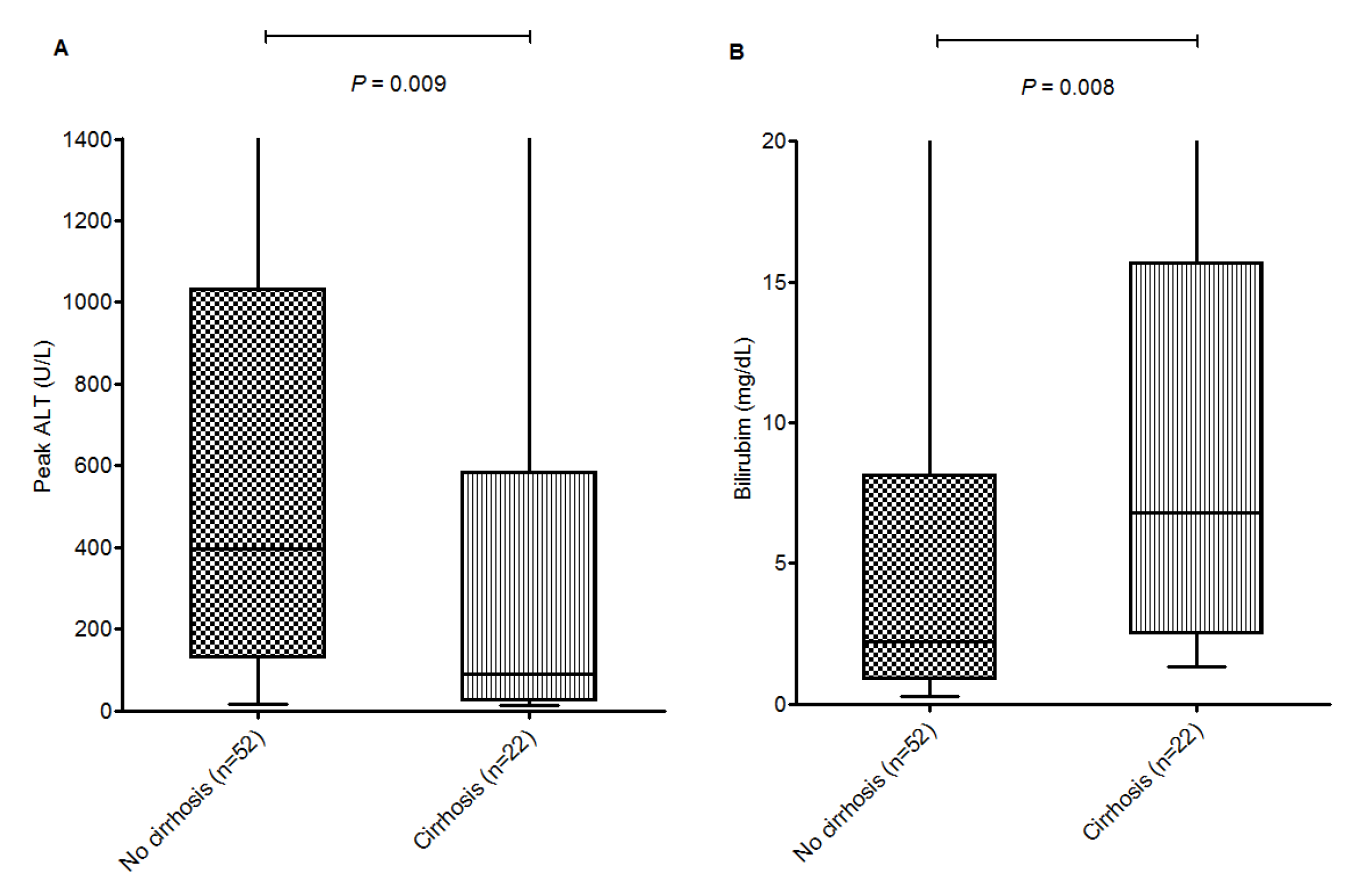

Supplement: Supplementary file 1 — Additional file 1. Fig. S1. [file 12879_2022_7050_MOESM1_ESM.tif]
