# Supplementary material for: Acute hepatitis E virus superinfection increases mortality in patients with cirrhosis
Source: BMC Infect Dis. 2022 Jan 18;22:62. doi: 10.1186/s12879-022-07050-w (PMC8767750; doi:10.1186/s12879-022-07050-w)
Supplement: Supplementary file 2 — Additional file 2: Table S1. Comparison of variables between survivors and non-survivors in HEV-infected patients (n = 74). Table S2. Comparison of variables between survivors and non-survivors in patients with cirrhosis (n = 22). Table S3. Predictive factors of 180-day mortality (n = 74). Table S4. Outcome in 7 patients with acute-on-chronic liver failure. [file 12879_2022_7050_MOESM2_ESM.docx]

Supp. Table 1. Comparison of variables between survivors and non-survivors in HEV-infected patients (n=74)

|  | Total | Survivors | Non-survivors | *P* |
| --- | --- | --- | --- | --- |
| No. | 74 (100%) | 67 (90.5%) | 7 (9.5%) |  |
| Age, year | 56.0 (42.8 - 68.0) | 55.0 (42.0 – 64.0) | 70.0 (44.0 – 76.0) | 0.075 |
| Male gender | 50 (67.6%) | 46 (68.7%) | 4 (57.1%) | 0.675 |
| Diabetes | 11 (14.9%) | 10 (14.9%) | 1 (14.3%) | 1.000 |
| Alcohol > 40 g/day | 20 (27.0%) | 18 (26.9%) | 2 (28.6%) | 1.000 |
| Hypertension | 13 (17.6%) | 11 (16.4%) | 2 (28.6%) | 0.599 |
| CKD | 2 (2.7%) | 2 (3.0%) | 0 | 1.000 |
| Malignancy | 12 (16.2%) | 10 (14.9%) | 2 (28.6%) | 0.317 |
| Clinical symptoms |  |  |  |  |
| Jaundice | 33 (44.6%) | 30 (44.8%) | 3 (42.9%) | 1.000 |
| Fatigue | 18 (24.3%) | 16 (23.9%) | 2 (28.6%) | 1.000 |
| Nausea/vomiting | 10 (13.5%) | 10 (14.9%) | 0 | 0.583 |
| Fever | 14 (18.9%) | 12 (17.9%) | 2 (28.6%) | 0.611 |
| Abdominal pain | 16 (21.6%) | 15 (22.4%) | 1 (14.3%) | 1.000 |
| No symptom | 14 (18.9%) | 14 (20.9%) | 0 | 0.334 |
| Undercooked meat | 14 (18.9%) | 14 (20.9%) | 0 | 0.334 |
| Laboratory data |  |  |  |  |
| WBC, ×*10^9^/L* | 6.1 (5.1 - 9.6) | 6.0 (4.9 – 9.5) | 6.7 (5.5 – 12.3) | 0.530 |
| Hemoglobin, *g/dL* | 13.4 (11.7 - 14.8) | 13.5 (12.2- 15.1) | 11.2 (9.0 – 13.6) | 0.024 |
| Platelet, ×*10^9^/L* | 177.5 (116.5 - 266.3) | 184.0 (119.0- 269.0) | 138.0 (72.0 – 159.0) | 0.037 |
| AST, *U/L* | 257.0 (72.5 - 868.3) | 259.0 (68.0 – 852.0) | 140.0 (74.0 – 917.0) | 0.561 |
| ALT, *U/L* | 347.5 (89.3 - 959.3) | 349.0 (109.0 – 1051.0) | 60.0 (23.0 – 508.0) | 0.076 |
| Creatinine, *mg/dL* | 0.82 (0.65 - 0.91) | 0.80 (0.65 – 0.89) | 1.11 (0.71 – 1.42) | 0.059 |
| Bilirubin, *mg/dL* | 4.3 (1.3 - 8.7) | 4.25 (1.19 – 9.24) | 4.35 (2.07 – 8.36) | 0.631 |
| Albumin, g/dL | 3.7 (3.4 - 4.1) | 3.8 (3.5 – 4.2) | 2.9 (2.4 – 3.7) | 0.003 |
| Sodium, mmol/L | 136.9 (134.4 - 139.5) | 137.4 (134.8 – 139.8) | 131.2 (128.1 – 136.2) | 0.002 |
| PT-INR | 1.13 (1.00 - 1.44) | 1.13 (1.00 – 1.23) | 1.54 (1.47 – 1.96) | 0.003 |

Abbreviation: CKD, chronic kidney disease; WBC, white blood cell; AST, aspartate transaminase; ALT, alanine transaminase; PT-INR, prothrombin time- international normalized ratio.

*P*: Mann-Whitney U-test and Chi-squared test.

Data are presented as the median (interquartile range) for continuous data and percentages for categorical data.

Supp. Table 2. Comparison of variables between survivors and non-survivors in patients with cirrhosis (n=22)

|  | Total | Survivors | Non-survivors | *P* |
| --- | --- | --- | --- | --- |
| No. | 22 | 17 | 5 |  |
| Age, year | 56.5 (47.0-68.0) | 56.0 (48.5 – 59.0) | 68.0 (43.5 – 73.0) | 0.446 |
| Male gender | 16 | 13 | 3 | 0.585 |
| Diabetes | 8 | 7 | 1 | 0.613 |
| Alcohol > 40 g/day | 9 | 7 | 2 | 1.000 |
| Hypertension | 4 | 2 | 2 | 0.210 |
| Malignancy | 3 | 3 | 0 | 1.000 |
| Jaundice | 13 | 11 | 2 | 0.609 |
| Undercooked meat | 4 | 4 | 0 | 0.535 |
| Laboratory data |  |  |  |  |
| WBC, ×*10^9^/L* | 6.4 (5.1 – 10.9) | 6.2 (4.7 – 9.3) | 6.7 (5.4 – 13.8) | 0.189 |
| Hemoglobin, *g/dL* | 13.0 (11.1 – 14.4) | 13.0 (11.3 – 14.7) | 11.2 (8.9 – 14.0) | 0.319 |
| Platelet, ×*10^9^/L* | 117.0 (83.3 – 159.8) | 119.0 (90.5 – 170.0) | 109.0 (61.5 – 155.5) | 0.493 |
| AST, *U/L* | 168.5 (55.0 – 1288.0) | 255.0 (54.0 – 1386.5) | 82.0 (56.5 – 866.5) | 0.880 |
| ALT, *U/L* | 90.0 (24.5 - 585.0) | 121.0 (25.5 – 876.5) | 45.0 (22.5 – 469.5) | 0.493 |
| Creatinine, *mg/dL* | 0.84 (0.65 – 0.93) | 0.84 (0.63 – 0.91) | 1.24 (0.75 – 1.63) | 0.850 |
| Bilirubin, *mg/dL* | 6.8 (2.5 – 15.7) | 6.3 (3.0 – 18.3) | 7.6 (2.2 – 18.4) | 0.940 |
| Albumin, g/dL | 3.5 (2.8 - 3.8) | 3.5 (2.9 – 3.9) | 2.9 (2.6 – 3.7) | 0.319 |
| Sodium, mmol/L | 134.1 (131.4 – 137.3) | 134.9 (132.7 – 138.7) | 131.2 (123.8 – 134.0) | 0.048 |
| PT-INR | 1.51 (1.19 – 2.02) | 1.47 (1.18 – 2.15) | 1.54 (1.33 – 1.80) | 0.940 |

Abbreviation: WBC, white blood cell; AST, aspartate transaminase; ALT, alanine transaminase; PT-INR, prothrombin time- international normalized ratio.

*P*: Mann-Whitney U-test and Chi-squared test.

Data are presented as the median (interquartile range) for continuous data and percentages for categorical data.

Supp. Table 3. Cirrhosis as risk factors for 180-day mortality

| Variable | *P* | HR (95% CI) |
| --- | --- | --- |
| Cirrhosis | 0.030 | 6.151 (1.192 – 31.726) |
| Cirrhosis (age)* | 0.018 | 8.095 (1.424 – 46.016) |
| Cirrhosis (age, sex)* | 0.018 | 8.111 (1.432 – 45.937) |

Abbreviation: HR, hazard ratio; CI, confidence interval.

*Cirrhosis corrected for mentioned parameters.

Supp. Table 4. Outcome in 7 patients with acute-on-chronic liver failure

| Age and sex | Precipitants | Type of organ failure | ACLF grade | CLIF-C ACLF score | Prognosis |
| --- | --- | --- | --- | --- | --- |
| 76 F | HEV infection | Hepatic + Coagulation failure | 2 | 55 | Death due to hepatic failure |
| 43 M | Bacterial infection | Respiratory + Cerebral + Circulatory failure | 3 | 54 | Death due to sepsis |
| 70 F | HEV infection | Kidney failure | 1 | 53 | Death due to hepatic failure |
| 48 M | Alcoholic hepatitis | Kidney failure | 2 | 51 | No death |
| 44 M | Alcoholic hepatitis | Hepatic + Kidney + Coagulation failure | 3 | 49 | Death due to hepatic failure |
| 43 F | HEV infection | Hepatic + Coagulation failure | 2 | 43 | No death |
| 56 F | Alcoholic hepatitis + Bacterial infection | Hepatic + Coagulation failure | 2 | 46 | No death |
